# Facile Synthesis of Eggshell Membrane-Templated Au/CeO_2_ 3D Nanocomposite Networks for Nonenzymatic Electrochemical Dopamine Sensor

**DOI:** 10.1186/s11671-019-3203-8

**Published:** 2020-01-29

**Authors:** Qingquan Liu, Xiaoyu Chen, Ze-Wen Kang, Chaohui Zheng, Da-Peng Yang

**Affiliations:** 1 Department of Pulmonary and Critical Care Medicine, The Second Affiliated Hospital of Fujian Medical University, Quanzhou, 362000 Fujian Province China; 2 College of Chemical Engineering and Materials Science, Quanzhou Normal University, Quanzhou, Fujian Province China

**Keywords:** Eggshell membrane, Au nanoparticles, CeO_2_, Electrochemical detection, Dopamine

## Abstract

**Abstract:**

Dopamine acts as a neurotransmitter to regulate a variety of physiological functions of the central nervous system. Thus, the fabrication of electrochemical active nanomaterials for sensitive dopamine detection is extremely important for human health. Herein, we constructed a highly efficient dopamine nonenzymatic biosensor using eggshell membrane (ESM) as a 3D network-like carrier-loaded Au and CeO_2_ nanocomposites. This approach has led to the uniform distribution of CeO_2_ and Au nanoparticles on the surface of ESM. The structure and properties of the as-prepared ESM templated Au/CeO_2_ (ESM-AC) nanocomposites were characterized. The electrochemical properties of non-enzymatic oxidation of dopamine by ESM-AC electrode were studied by cyclic voltammetry (CV) and differential pulse voltammetry (DPV). The detection limit of the ESM-AC modified electrode for dopamine is 0.26 μM with a linear range from 0.1 to 10 mM. The ESM-AC-modified electrode performs a higher catalytic activity for dopamine electrocatalytic oxidation than that ESM-templated CeO_2_ (ESM-C) electrode, which is mainly due to the unique structure of ESM and more active sites provided from Au. Collectively, this biological waste-ESM provides a cheap and unique template for the preparation of 3D network-like nanostructures and expands the application in electrochemical dopamine detection.

**Graphical abstract:**

ESM-AC nanocomposites prepared from biological waste was successfully modified on the surface of glassy carbon electrode and a dopamine-based electrochemical biosensor was constructed. 
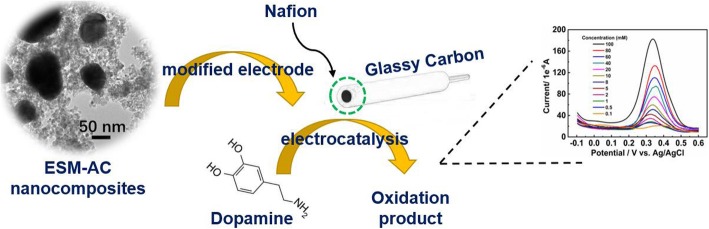

## Introduction

Dopamine is a neurotransmitter to help cells transmit pulsed chemicals. Scientific research has also shown that various addictive behaviors are closely related to dopamine [1]. The content of dopamine in the body is directly related to life and health, so the establishment of rapid, efficient, and extensive dopamine detection method is of great importance in practical applications [2]. The commonly used methods for determination of dopamine include fluorescence analysis [3, 4]. enzyme-linked method [5], gas chromatography [6], and high-performance liquid chromatography [7]. Although effective detection methods for dopamine have been successfully established, most of these methods require specific biological enzymes or complex detection processes [8–10]. However, the high sensitivity inherent in electrochemical methods and the high enzyme-free specificity that has emerged in recent years have made electrochemical biosensors a promising dopamine detection tool, and the defects in biological enzyme activity and stability depending on environmental conditions (such as temperature, pH and immobilization strategy) could be greatly avoided [11–14].

Cerium oxide is a non-inert carrier of a commonly used catalyst which simultaneously exists in Ce^3+^ and Ce^4+^ oxidation states on the lattice surface and is currently used in many fields such as analysis detection, drug delivery, and photocatalysis [15]. In the past few years, great progress has been made in scientific research around CeO_2_ [16]. For example, Shi and colleagues developed a mesostructured CeO_2_/g-C_3_N_4_ nanocomposite to significantly improve the photocatalytic activity of CO_2_ by activation of mutual components [17]. Yu and co-workers demonstrated that CeO_2_/CoSe_2_ nanobelt composite had an efficient electrochemical water oxidation action. The unique surface structure of CeO_2_ with high mobility of oxygen vacancies in nanoscale helped enhance the oxygen evolution reaction (OER) activity [18]. However, some scientific research has found that the redox signal of CeO_2_ can be detected in neutral mild buffer solutions at about 0.45 V, which means the signal is too weak to be used in biosensors [19]. Therefore, it is very important to find a way to enhance the CeO_2_ redox signal to construct a highly sensitive biosensor. In another work, Chai and colleagues established the Cu/Mn double-doped CeO_2_ nanocomposites to enhance the sensitive electrochemical detection of procalcitonin, which exhibited a more excellent catalytic activity than that pure CeO_2_ [19].

Recently, the hierarchically porous structure of natural biological templates and their composite materials have attracted wide attention due to their unique properties and wide application prospects [20–22]. As an agricultural waste, it is estimated that more than 7.0 million tons of eggshell waste in the world were produced every year [23]. ESM is one of the most widely used natural biological templates, and its unique 3D network structure provides its natural advantages with low cost and non-toxicity as a supporting template [24], compared to other biological templates, such as bacterial virus templates [25], algae templates [26], plant templates [27], and DNA templates [28]. The main advantage of ESM is that it has a wide range of sources, low cost, no pollution to the environment, and simple separation and purification process [29]. For example, Zhong et al. established a high-performance asymmetric supercapacitor bioreactor based on the 3D porous carbon network of ESM; this work adequately summarizes the advantages of ESM in electrochemical research, such as ESM can be used as a high-conductivity path. The microporous structure in ESM can effectively reduce the diffusion resistance of ions, and the 3D network structure had good structural stability, which can improve the stability of composite materials and increase the number of cycles [30].

Herein, we utilized the most common biowaste ESM to construct a 3D porous network CeO_2_ and Au/CeO_2_ biosensor for electrochemical catalytic oxidation of dopamine. In the as-synthesized nanocomposite, CeO_2_ was uniformly distributed on the surface of the ESM and Au is dispersed and embedded in the form of particles. This unique 3D structure provides more contact area for electrochemical reactions and enhances mass transfer during electrochemical reactions. In addition, we used CV and DPV to carry out nonenzymatic dopamine sensing studies on ESM-C and ESM-AC modified electrodes. Both electrodes exhibited good electrochemical reactivity and selectivity. Below is the detail.

## Results and Discussion

### Characterization

**Fig. 1:**
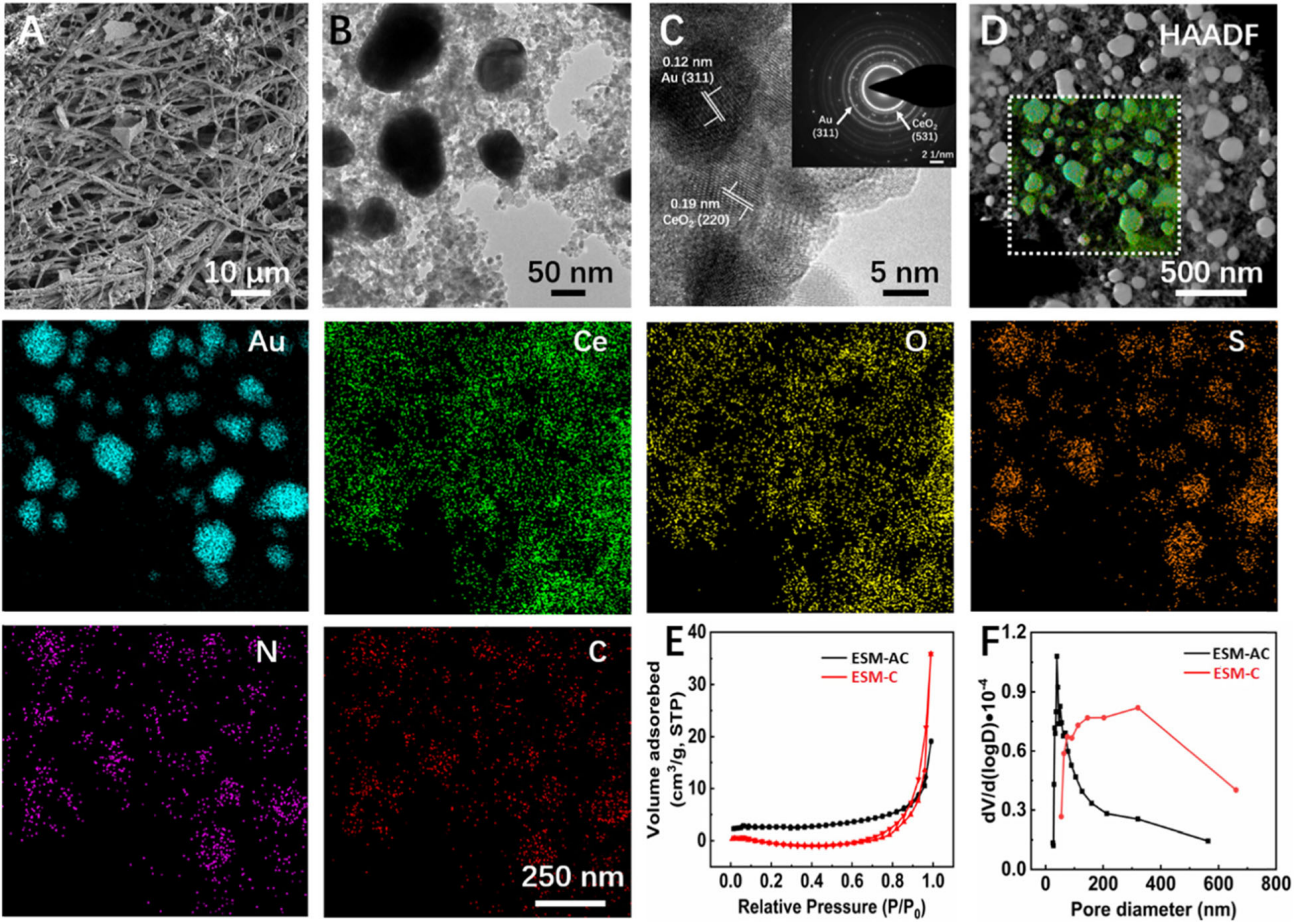
Characterization showing the morphological attributes of as-prepared ESM-AC nanocomposites. (**a**) FE-SEM; (**b**) TEM images; (**c**) high-resolution TEM, and the inset showing the selected-area electron diffraction (SAED); (**d**) TEM-based elemental mapping; (**e**) N_2_ adsorption−desorption isotherms of ESM-C and ESM-AC; and their (**f**) corresponding pore size distribution curves obtained by the BJH method

The morphology and surface structure of ESM-C and ESM-AC nanocomposites were characterized by FE-SEM (Fig. 1(a)) and HR-TEM (Fig. 1(b)). In the FE-SEM image, the 3D network structure of the ESM vertical and horizontal alternating and the large number of nanoparticles successfully loaded on the surface can be clearly observed. To further certify the materials composition of the ESM surface, the ESM-C and ESM-AC nanocomposites were characterized by HR-TEM respectively. Additional file 1: Figure S1A shows the TEM image of ESM-C nanocomposites. Combined with the distribution of elements in the TEM mapping (high-angle annular dark field-HAADF, Additional file 1: Figure S1B), it can be seen that CeO_2_ was uniformly distributed on the surface of ESM. Similarly, in the HAADF characterization of ESM-AC nanomaterials (Fig. 1(d)), CeO_2_ was also uniformly distributed on the surface of ESM, and the Au nanoparticles were uniformly distributed in the material mainly at a particle size of 60-90 nm. In addition, the HAADF test results showed that the elemental signals of strong Au and Ce detected were significantly stronger than the C and N element signals in the ESM itself. This result clearly demonstrated that the 3D network-like structure was mainly composed of Au and CeO_2_. Moreover, The HR-TEM image (Fig. 1(c)) exhibited the lattice stripe-like structures of the ESM-AC, which were uniform with the spacings of 0.12 and 0.19 nm, corresponding to (311) and (220) crystal surfaces of Au and CeO_2_, respectively. And the selected-area electron diffraction (SAED) pattern (Fig. 1(c inset)) in the selected region showed the polycrystalline character and was in agreement with the subsequent XRD results. To further verify the properties of the pore structure, we measured the finer surface properties of the ESM-C and ESM-AC nanocomposites by measuring the specific surface area based on the N_2_ adsorption−desorption isotherms using Brunauer−Emmett−Teller (BET) method and the Barrett−Joyner−Halenda (BJH) method based on the adsorption curve to obtain the corresponding pore size distribution (Fig. 1(e, f)). Based on the International Union of Pure and Applied Chemistry (IUPAC) classification, the BET isotherm of the resulting ESM-C nanocomposite (Fig. 1(e)) showed a typical IV-type adsorption mode with a hysteresis loop indicating a typical surface characteristic of porous materials and a desorption cumulative pore volume of 0.06 cm^3^ g^−1^. However, the BET isotherm of ESM-AC nanocomposites resulted in a rare III-type isotherm with a concave-shaped and no inflection point, and the desorption cumulative pore volume is 0.03 cm^3^ g^−1^. Interestingly, the specific surface area of ESM-AC (S_BET_ = 8.9005 m^2^ g) was nearly 1.8 times greater than that of ESM-C (S_BET_ = 4.9827 m^2^ g). This result indicated that ESM-AC was better able to make contact with the sample of the analyte when it was electrochemically reacted in the solution.

**Fig. 2:**
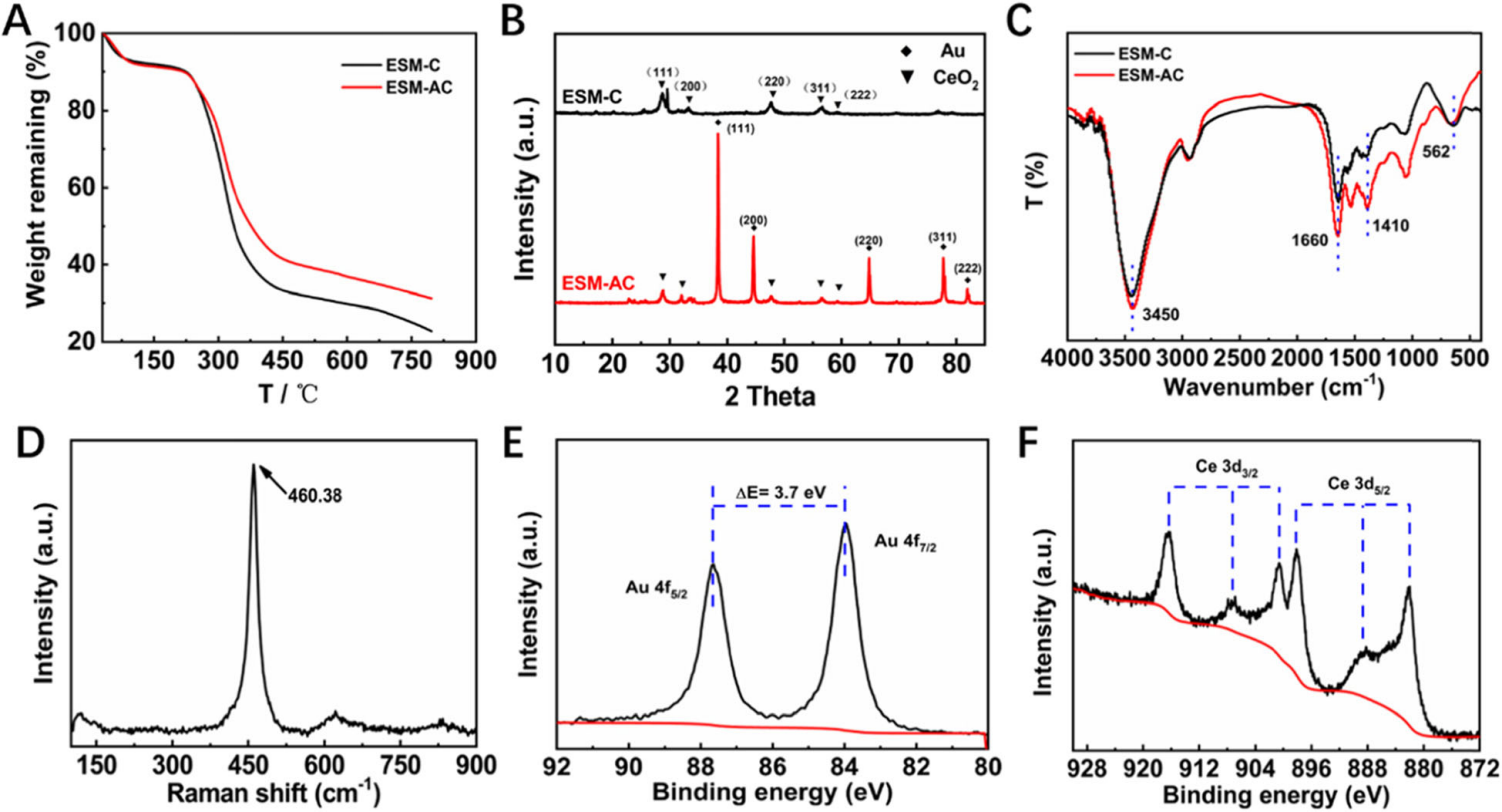
Characterization of ESM-C and ESM-AC nanocomposites. **a** TGA analysis. **b** XRD analysis. **c** FT-IR spectra. **d** Raman spectra of ESM-AC. **e**, **f** XPS analysis of ESM-AC **e** Au 4f and **f** Ce 3d

Moreover, to further understand and explore the intrinsic properties and structural characteristics of the two nanocomposites, we used thermogravimetric analysis (TGA) to characterize the thermal stability and thermal reaction of the materials. As shown in Fig. 2a, the two nanocomposites both exhibited two distinct weight loss processes. The weight loss process at around 100 °C was mainly caused by the evaporation of water molecules in the materials and the weight loss process from 250 to 500 °C was mainly based on the carbonization of organic matter in the nanocomposite to form CO_2_. Therefore, in the preparation process, after the precursors of ESM-C and ESM-AC were constructed by using the natural 3D network structure of the ESM, the precursor was chosen to calcine at 550 °C which allowed the ESM to be completely decomposed. It was obvious that the final mass residue of ESM-AC was significantly higher than that ESM-C, which was mainly based on the quality of Au nanoparticles. Furthermore, the purity and existing phase of the ESM-C and ESM-AC were characterized by XRD patterns. As shown in Fig. 2b, the diffraction peaks of CeO_2_ and Au in both nanocomposites can be clearly detected, and the diffraction angles 2*θ* at 28.549°, 33.083°, 47.486°, 56.346°, and 59.093° respectively corresponding to (111), (200), (220), (311), and (222) CeO_2_ crystal planes, conforming to the standard diffraction peak of face-centered cubic fluorite-type CeO_2_ (JCPDS NO. 75-0120). Similarly, the 2*θ* at 38.187°, 44.385°, 64.576°, 77.566°, and 81.722° respectively correspond to the (111), (200), (220), (311), and (222) crystal planes of Au, which are in accordance the standard diffraction peak (JCPDS NO. 99-0056). These indicated that we have successfully prepared the samples. It was noted that the peak intensity of CeO_2_ and Au in the XRD test results were obvious, which further indicated that the content of CeO_2_ and Au in the samples were significant. This was also consistent with the HAADF results. FT-IR and Raman analysis were studied to identify the molecular configuration of ESM-C and ESM-AC nanocomposites, which are displayed in Fig. 2c and d. The FT-IR spectra (Fig. 2c) of these two samples exhibited wavenumber at 4000—400 cm^−1^, being typical stretching vibration or bending vibration of some functional groups [31]. The peak at about 3450 and 1660 cm^−1^ region were due to the O–H stretching vibration and bending vibration of water molecules adsorbed by the two samples, respectively. The minor peak at 1410 cm^−1^ could be attributed to the CO_2_, which is usually the nanocrystalline materials that absorbed from the air environment due to their high surface to volume ratio. Another absorption band at around 562 cm^−1^ was the characteristic peak for the Ce-O stretching vibration [32]. However, the FT-IR spectra of ESM-AC had little difference from those of ESM-C, probably because the fact that some of the absorption peaks were changed after the ESM-AC material was reduced by NaOH during the preparation process. To be more convincing, the structure of CeO_2_ in the two nanocomposites was further clarified by Raman spectra, as shown in Fig. 2d. The nanocomposites showed a strong band at 460.38 cm^−1^, which was basically consistent with the F_2g_ Raman activation-mode of the fluorite-type cubic structure [32, 33]. Raman spectra once again confirmed the composition of the synthesized product and its good crystal structure.

Furthermore, the surface electron state and chemical composition of the synthesized ESM-AC were demonstrated by XPS analysis (Fig. 2e, f). As can be seen in Fig. 2e, the Au 4f peak can be fitted into two peaks at 84.0 (4f_7/2_) and 87.7 eV (4f_5/2_) for ESM-AC sample, which can be attributed to Au^0^ metal [34]. Similarly, the high-resolution Ce 3d XPS spectrum of the ESM-AC nanocomposites in Fig. 2f contained six obvious peaks, and they could be resolved into several Ce 3d_5/2_ and Ce 3d_3/2_ spin–orbit doublet peaks with splitting of around 18.6 eV. Ce^3+^ and Ce^4+^ spectra have different multiple splitting, Ce^4+^ has peak at 917 eV which is absent in Ce^3+^ spectrum [35, 36]. Meanwhile, XPS spectra of ESM-C nanocomposites were also tested for comparison (Additional file 1: Figure S2A-B). In Additional file 1: Figure S2A, the valence of Ce has a combination of +3 and + 4, indicating the presence of Au can better help Ce form a +4-valence state. The full XPS spectra of ESM-AC nanocomposites are shown in Additional file 1: Figure S3.

### Electrochemistry Activity Towards Dopamine Oxidation in Different Media

**Fig. 3:**
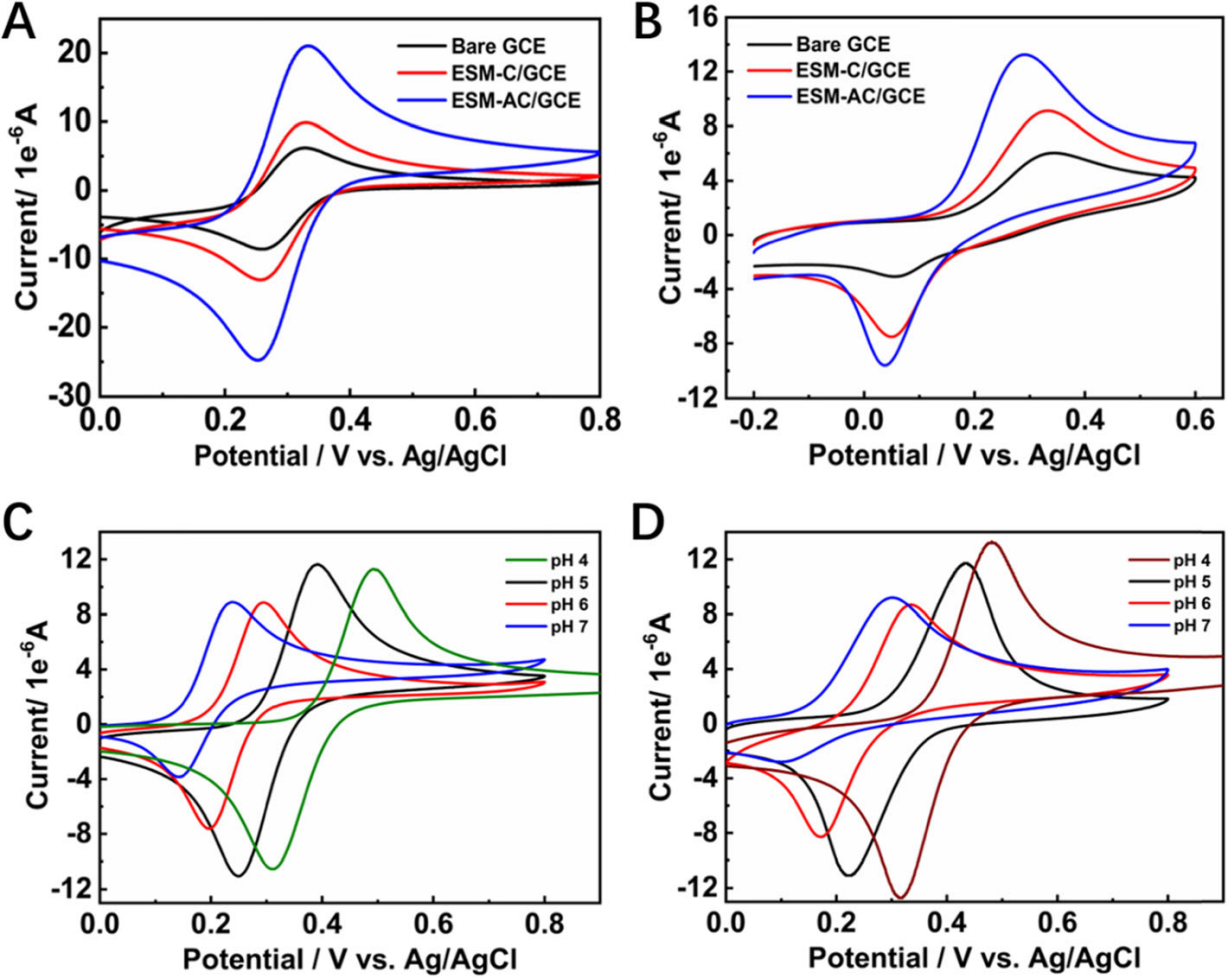
CVs for different electrodes recorded in **a** 0.5 mM [Fe(CN)_6_]^3−/4−^. **b** 1 mM dopamine with 0.2 M PBS. **c** ESM-C electrode in 1 mM dopamine with various pH. **d** ESM-AC electrode in 1 mM dopamine with various pH at a scan rate of 50 mV s^−1^

The nonenzymatic oxidation of dopamine at unmodified bare GCE, ESM-C/GCE, and ESM-AC/GCE electrodes (Additional file 1: Figure S4) was studied using CV and DPV, and their electrochemistry activity characteristics were compared. In the present of 0.5 mM [Fe(CN)_6_]^3−/4−^ at a scan rate of 50 mV s^−1^ system, the CV method can be selected as a standard step before and after the processing behavior of the labeled electrode. As shown in Figure 3a, there was reversible redox response for bare GCE electrode in the presence of [Fe(CN)_6_]^3−/4−^, while for ESM-C/GCE and ESM-AC/GCE modified electrodes, the redox peaks currently increased greatly due to increasing effective surface area and conductivity in the presence of the ESM-C and ESM-AC nanocomposites. Therefore, we then examined the response of different electrodes in the dopamine detection system. In the [Fe(CN)_6_]^3−/4−^ system, the GCE electrodes modified with ESM-C and ESM-AC nanocomposites both exhibited strong redox ability, which showed the promotion of electrochemical catalytic oxidation of the two nanocomposites [37]. Unexpectedly, the same electrochemical catalytic oxidation results were also evident in the dopamine detection system shown in Fig. 3b. The GCE electrodes modified with ESM-C and ESM-AC nanocomposites both exhibited a significantly enhanced redox capacity for dopamine relative to the unmodified bare GCE electrode. Among them, the response applied potential of ESM-AC modified GCE electrode to dopamine electrochemical catalytic oxidation was significantly lower than ESM-C modified GCE (relatively lower 0.043 V) and unmodified bare GCE electrode (relatively lower 0.052 V), and a high sensitivity was easily achieved. To further investigate the electrochemical catalytic oxidation of dopamine by ESM-C and ESM-AC modified GCE electrodes, we explored the CV response of different pH systems. Figure 3c, d displays the CV response for ESM-C/GCE and ESM-AC/GCE in various pH systems with 1 mM dopamine at a scan rate of 50 mV·s^−1^, respectively. Interestingly, the current response applied potential were significantly decreased when an increased value of pH was performed, indicating a higher pH buffer system can reduce the response voltage of the two electrodes to dopamine electrochemically catalyzed oxidation. However, as the pH increases, the electrochemical catalytic oxidation activity of the two electrodes on dopamine also decreases, so the buffer system cannot be selected only according to the response potential [38]. Considering that the response potential at pH 4.0 is acceptable and the activity of electrochemically catalyzing the oxidation of dopamine is relatively higher, the buffer system of the subject finally selected pH 4.0 as the optimum pH value.

**Fig. 4:**
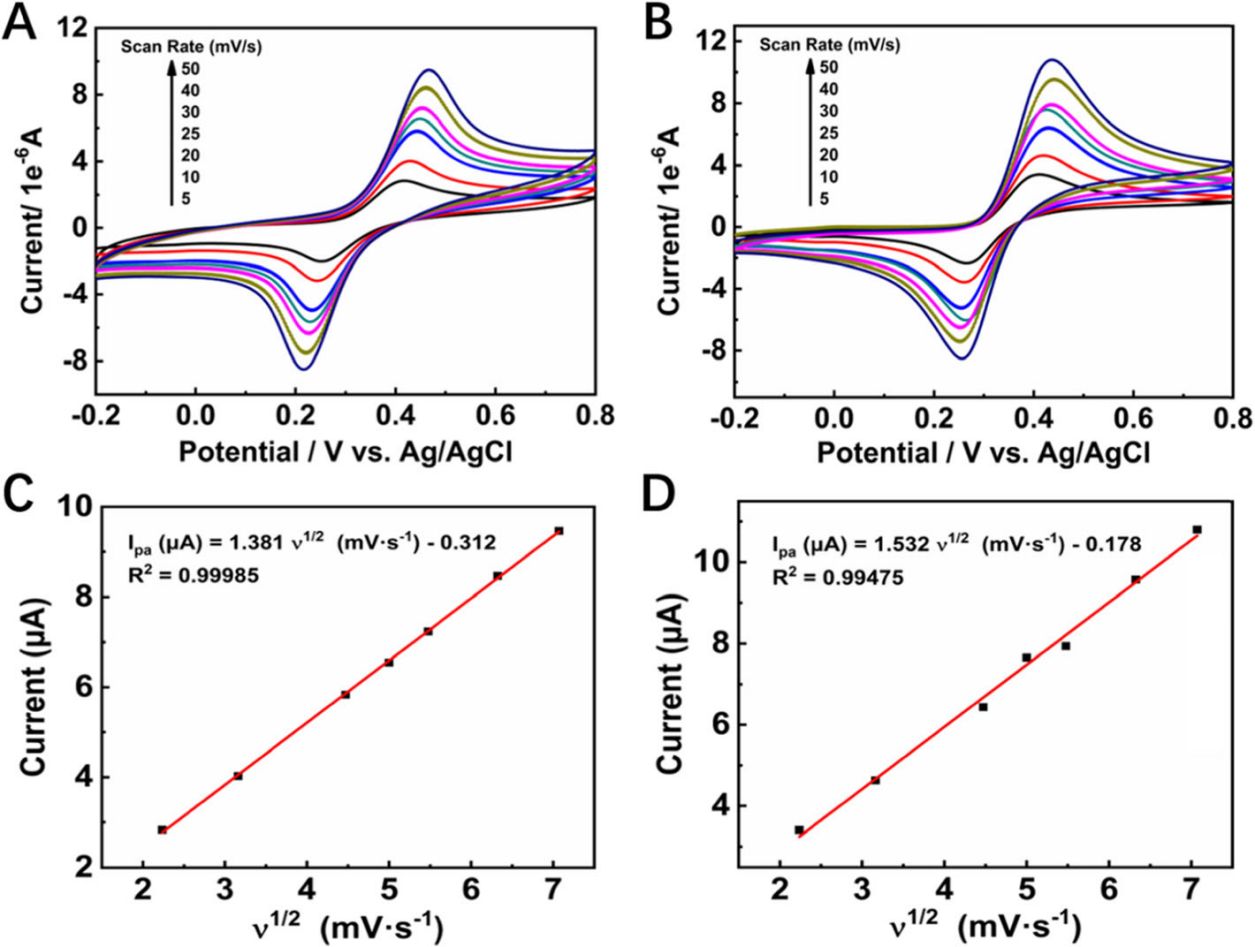
CV curves of the **a** ESM-C- and **b** ESM-AC-modified electrodes in 0.2 M PBS (pH 4.0) containing 1 mM dopamine at varied scan rates (5–50 mV s^−1^), and **c**, **d** the corresponding correlations between the peak current and scan rate

Furthermore, CV measurements were recorded on ESM-C/GCE and ESM-AC/GCE electrodes with dopamine (1 mM) analytes at different scan rates (5–50 mV s^−1^, Fig. 4a, b) with pH 4.0. As the scan rate was from low to high (5–50 mV s^−1^), the redox peak current increases, and the reversible reduction peak potential also moves in a more negative direction. This result indicated that electrochemical reduction of dopamine on the surface of ESM-C/GCE and ESM-AC/GCE was a reversible process. Figure 4c, d exhibits the reversible anodic peak current, which is proportional to the square root of scan rate with a linear regression equation for ESM-C which could be expressed as *I*_pa_ (μA) = 1.381ν^1/2^ (mV s^−1^) − 0.312 and a correlation coefficient (*R*^2^) of 0.99985, for ESM-AC, which could be expressed as *I*_pa_ (μA) = 1.532ν^1/2^ (mV s^−1^) − 0.178 and a correlation coefficient (*R*^2^) of 0.99457. Those results in Fig. 4c, d clearly indicate that the ESM-C- and ESM-AC-modified GCE electrodes both were the desirable nanocomposite materials for electrochemical redox of dopamine with a typical diffusion-controlled process occurs [39]. Based on these results, it was clear that the as-prepared ESM-C and ESM-AC nanocomposites had excellent catalytic activity towards dopamine electrocatalytic oxidization.

**Fig. 5:**
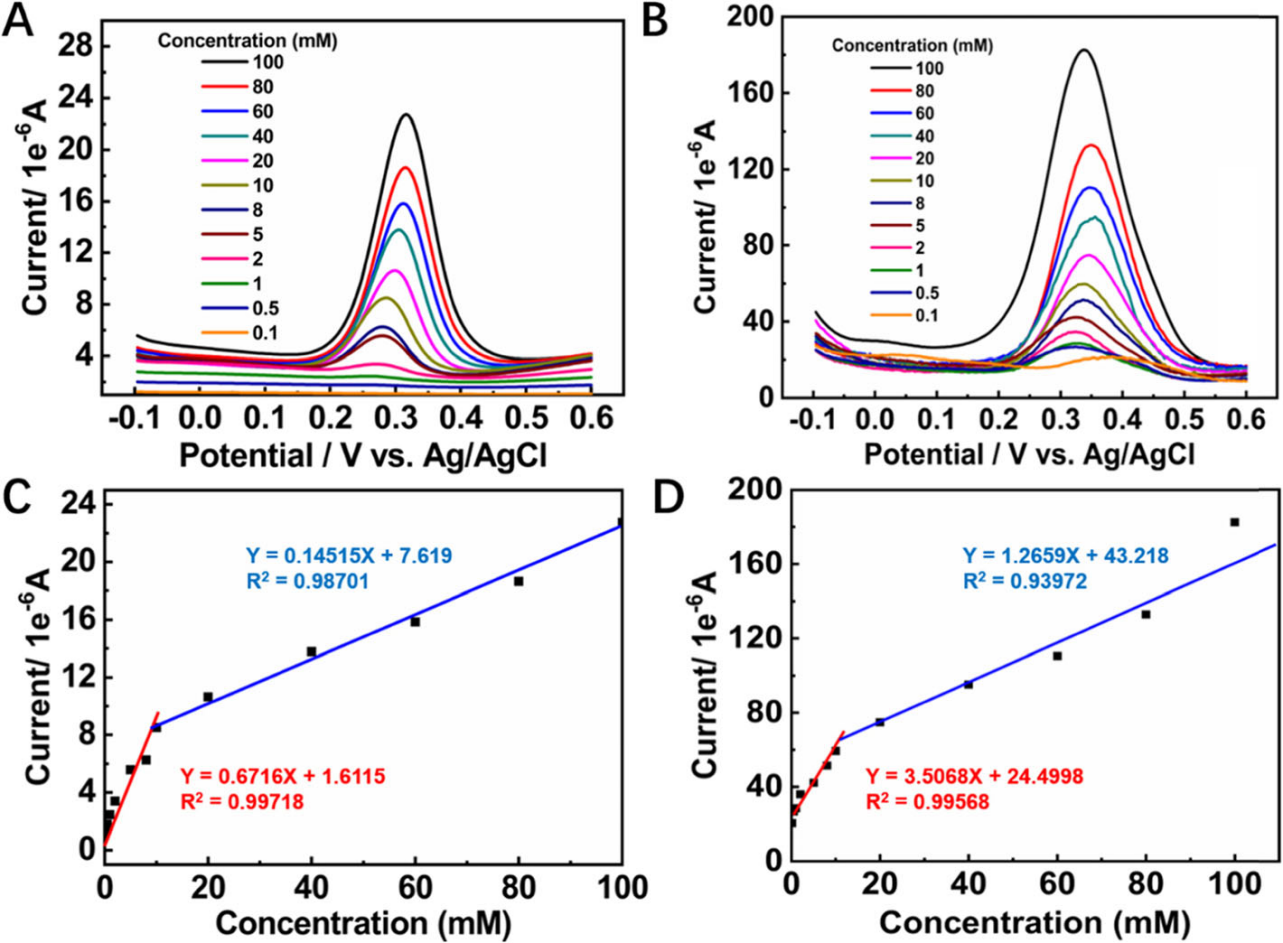
DPV response of the **a** ESM-C- and **b** ESM-AC-modified electrodes under consecutive addition of dopamine within a total dosage range of 0.1–100 mM, and **c**, **d** the corresponding calibration plot of response current with dopamine concentration

The redox properties of dopamine allow DPV to detect it more sensitively because the DPV can eliminate the non-Faradaic current. By holding the buffer solution at pH 4.0, the DPV responses of the ESM-C- and ESM-AC-modified GCE electrodes towards the different concentrations of dopamine were investigated. The anodic peak current (*I*_pa_) increased with the dopamine concentration in the range from 0.1 to 100 mM for both of the ESM-C- and ESM-AC-modified GCE electrodes (Fig. 5a, b) and had an excellent linear relationship with the dopamine concentrations (Fig. 5c, d). The sensitivity of DPV can be obtained by linear regression equation, and the ESM-AC/GCE system exhibited a much higher sensitivity of 3.5068 μA mM^−1^ with the linear range from 0.1 to 10 mM (*I*_pa_ (μA) = 3.5068 C (mM) + 24.4998 (*R*^2^ = 0.99568)) than the ESM-C/GCE system (*I*_pa_ (μA) = 0.6716 C (mM) + 1.6115 (*R*^2^ = 0.99718)).

The limit of detection (LOD) of electrochemical redox dopamine estimated at a LOD = 3*σ*/*N* formula, where *σ* is the standard deviation of blank and *N* is the slope of linear. Obviously, LOD of the ESM-AC/GCE-based electrochemical dopamine sensor (LOD = 0.26 μM in the range of 0.1 to 10 mM) was much lower than that ESM-C/GCE-based (LOD = 1.3 μM in the range of 0.1 to 10 mM). As mentioned before, such a high sensitivity could be attributed to the unique 3D hierarchical porous structure of ESM and Au nanoparticles, which provided a large surface area, plentiful active sites, and high conductivity and allowed the migration of dopamine to electrode with small hindrance. Furthermore, another calibration linear range was also found, which was from 20 to 100 mM with a linear regression equation of *I*_pa_ (μA) = 1.2659 C (mM) + 43.218 (*R*^2^ = 0.93972). The further increase of dopamine concentrations resulted in a gradual current saturation.

**Fig. 6:**
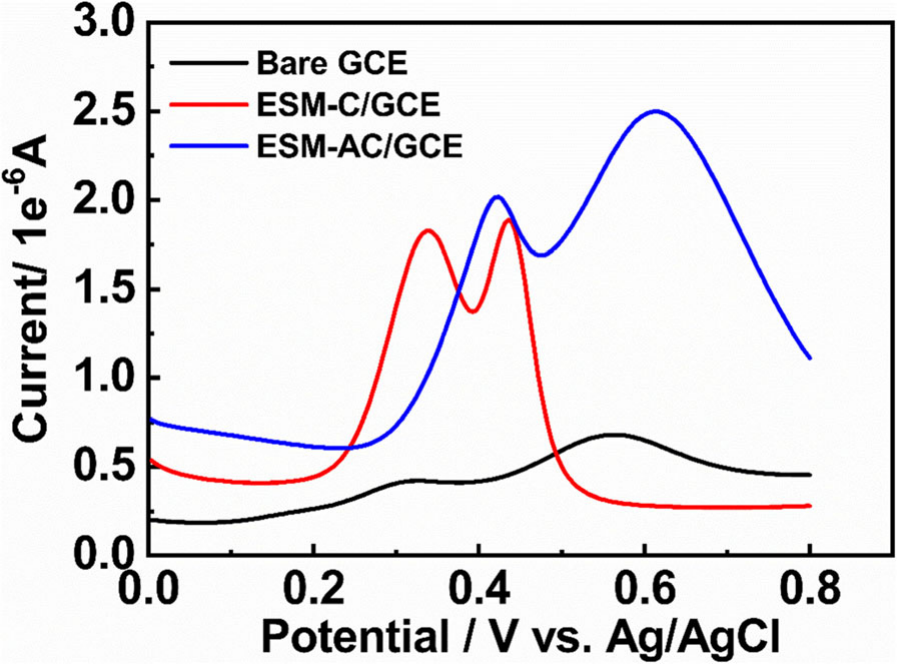
DPV of different electrodes under the conditions of dopamine and UC

The selectivity of the ESM-C/GCE and ESM-AC/GCE electrodes towards dopamine was also studied. In a mixed solution containing dopamine and UA, the similar oxidation potentials to dopamine were often present, making it difficult to quantify dopamine selectively. The results of DPV electrochemical catalytic oxidation with 20 μM dopamine and 20 μM UC are shown in Fig. 6. The results indicated that the two electrodes modified by ESM-C and ESM-AC nanocomposites had better peak shapes when detecting analytes, and the oxidation peaks of the two substances do not interfere with each other, while the unmodified bare electrode did not exhibit significant oxidation peak, indicating that the electrode modified by ESM-C and ESM-AC nanocomposites had better selectivity. The mechanism that ESM-AC with improved specificity could be attributed to the net-like 3D structure of eggshell membrane, a larger specific surface area, and a large number of C elements combined with Au elements increased the conductivity of the material, which made the electrochemical detection performance more outstanding. Furthermore, the analytical performances of our material were compared with some reported ones in terms of linear range and LOD. ESM-AC nanocomposites exhibited the lower LOD, as shown in table S1. In addition, our material also provides a potential application for simultaneous detection of dopamine and UC by ESM-C/GCE or ESM-AC/GCE.

## Conclusions

In summary, we successfully fabricated 3D network-like nanocomposites using eggshell membrane as template. These 3D network-like nanomaterials provided more active sites and can be used as an alternative material for nonenzymic dopamine sensor. Through investigating the relationship between scanning speed and current in CV, it was finally shown that both ESM-C- and ESM-AC-modified electrodes were diffusion-controlled processes for dopamine electrochemical catalytic oxidation. The LOD of the ESM-AC-modified electrode was 0.26 μM with a linear range from 0.1 to 10 mM, which was much lower than that ESM-C-modified electrode (LOD = 1.3 μM, linear range from 0.1 to 10 mM). Such a lower LOD of dopamine may be attributed to the migration of dopamine to electrode with small hindrance that based on the unique 3D-like network hierarchical structure of ESM and Au nanoparticles modified on GCE. Moreover, the electrode modified by ESM-C and ESM-AC nanocomposites towards dopamine electrochemical sensing exhibited good selectivity. Together, we believe that these electrodes based on ESM-modified nanocomposites may be used as a candidate for biomedical and clinical diagnosis [40].

## Methods Section

### Work Strategy

We compounded the gold nanoparticles with the biowaste ESM as a 3D network-like carrier and modified the resulting composite material on a glassy carbon electrode to construct a dopamine sensor for the disease prevention.

### Materials

Dopamine, uric acid (UA), HAuCl_4_·4H_2_O, Ce(NO_3_)_3_, and Nafion® perfluorinated resin solution (5 wt% in mixture of lower aliphatic alcohols and water) were purchased from Sigma-Aldrich. ESM was obtained mainly from the eggshells collected from local restaurants. All other chemicals used in this work were analytically pure (AR) and used without any further purification. Ultrapure water was used in all experiments and obtained from a Millipore water purification system (18.2 MΩ cm^−1^, Milli-Q).

### Instruments and Characterization

To investigate the surface morphology, field-emission scanning electron microscopy (FE-SEM, ZEISS Merlin Compact, Germany)-assisted energy dispersive X-ray spectroscopy (EDS, OXFORD 51-XMX, UK) and high-resolution transmission electron microscopy (HR-TEM, FEI Tecnai G2 F30, USA) were used. Further, the crystalline patterns of the material were tested by using the X-ray diffraction (XRD, D8 Advance, Bruker AXS, Germany). X-ray photoelectron spectroscopy (XPS, Thermo ESCALAB 250Xi, USA) was used to analyze the chemical valence of the elements. The chemical functionalities and structures of the designed polymeric nanoreactors were explored by Fourier-transform infrared spectroscopy (FT-IR, Thermo a Nicolet, USA) and a laser confocal Raman spectrometer (Renishaw, inVia, UK). Nitrogen adsorption−desorption isotherms were recorded using Micrometric (ASAP 2020, USA), and the Barrett−Joyner−Halenda (BJH) was used to estimate the pore size distributions of the as-prepared samples.

### Preparation of ESM-C and ESM-AC Nanocomposite Material

First, the ESM membrane pretreatment is done, which immerse the eggshell in ultra-pure water for 1 h to soften the inner surface membrane. Then, the ESM was dried in a bio-oven at 75 °C for 3 h and put 2.0-g dried ESM into 240-mL ultra-pure water and 30-mL anhydrous ethanol, and we stirred it for 1 h to ensure that the ESM was completely immersed in the solution. After that, the impregnated ESM was removed and macerated with 0.05 mM Ce(NO_3_)_3_ for 200 min. After soaking, the ESM was firstly cleaned with ultra-pure water and then cleaned with anhydrous ethanol for a second time and finally cleaned with ultra-pure water until the surface of the ESM was neutral. Then, it was dried in the oven at 60 °C for 30 min. Finally, the ESM cooled to room temperature and was calcined in the muffle furnace for 2 h at a temperature of 550 °C (heating rate of 2 °C min^−1^). Based on the successful synthesis of the ESM-C nanocomposites, the different process for the preparation of ESM-AC nanocomposites was after cleaning steps, which were repeated until the surface of the ESM was neutral, and then soaked with 30 mL of 0.01 M HAuCl_4_·4H_2_O for 30 min, and the above cleaning steps were repeated again. After that, 15 mL of 0.5 M NaOH was added to reduce the Au^3+^ attached to the ESM, and the ESM was cleaned again until it was neutral. The following preparation process was consistent with ESM-C nanocomposites.

### Preparation of Nano-inorganic Composite-Modified Electrode

Before modifying the electrode, the electrode surface was polished with alumina polishing powder (1.0 micron and 0.05 micron, respectively) on the polishing cloth and then shocked the electrode surface with deionized water and anhydrous ethanol alternately for about 5 min in the ultrasonic instrument, so as to achieve the purpose of removing impurities on the surface. Then, we took 5 mL of 5 mg mL^−1^ ESM-C suspension and dropped it on the electrode surface and let it dry naturally at room temperature. Moreover, we took 5 μL diluted Nafion solution with a concentration of 0.1% on the electrode surface and waited for it to dry naturally before conducting electrochemical experiments. The preparation of ESM-AC inorganic composite modified electrode was the same except that the modified material on the electrode surface was different.

### Electrochemical Detection of Dopamine

In this experiment, all electrochemical measurements were performed on a CHI 660E Electrochemical Workstation (Shanghai CH Instruments Inc., China) by using a conventional three-electrode system, the modified (loaded ESM-C or ESM-AC) glassy carbon electrode (GCE) was used as the working electrode, platinum wire electrode as the auxiliary electrode, and Ag/AgCl electrode as the reference electrode. 0.2 mol L^−1^ Na_2_HPO_4_ and 0.2 mol L^−1^ NaH_2_PO_4_ were used as the base solution to configure phosphate buffer solution (PBS) for electrochemical detection of dopamine.

## Supplementary information

**Additional file 1: Figure S1.** (A) TEM image of ESM-C nanocomposites; (B) TEM-based elemental mapping of ESM-C nanocomposites. **Figure S2.** XPS spectra of ESM-C nanocomposites: (A) Ce 3d and (B) O 1 s. **Figure S3.** Full XPS spectra of ESM-AC nanocomposites. **Figure S4.** FE-SEM images of ESM (A), ESM-C and ESM-AC on the surfaces of electrodes. **Table S1.** The analytical performances of various materials for the detection Dopamine.

## Data Availability

All data and materials are fully available without restriction.

## Abbreviations

BET: Brunauer−Emmett−Teller
BJH: Barrett−Joyner−Halenda
CV: Cyclic voltammetry
DPV: Differential pulse voltammetry
EDS: Energy dispersive X-ray spectroscopy
ESM: Eggshell membrane
ESM-AC: ESM templated Au/CeO2
ESM-C: ESM templated CeO2
FE-SEM: Field-emission scanning electron microscopy
FT-IR: Fourier-transform infrared spectroscopy
GCE: Glassy carbon electrode
HR-TEM: High-resolution transmission electron microscopy
LOD: Limit of detection
OER: Oxygen evolution reaction
PBS: Phosphate buffer solution
SAED: Selected-area electron diffraction
UA: Uric acid
XPS: X-ray photoelectron spectroscopy
XRD: X-ray diffraction
