# Supplementary material for: Facile Synthesis of Eggshell Membrane-Templated Au/CeO2 3D Nanocomposite Networks for Nonenzymatic Electrochemical Dopamine Sensor
Source: Nanoscale Res Lett. 2020 Jan 29;15:24. doi: 10.1186/s11671-019-3203-8 (PMC6989712; doi:10.1186/s11671-019-3203-8)
Supplement: Supplementary file 1 — Additional file 1: Figure S1. (A) TEM image of ESM-C nanocomposites; (B) TEM-based elemental mapping of ESM-C nanocomposites. Figure S2. XPS spectra of ESM-C nanocomposites: (A) Ce 3d and (B) O 1 s. Figure S3. Full XPS spectra of ESM-AC nanocomposites. Figure S4. FE-SEM images of ESM (A), ESM-C and ESM-AC on the surfaces of electrodes. Table S1. The analytical performances of various materials for the detection Dopamine. [file 11671_2019_3203_MOESM1_ESM.docx]

Supporting Information

**Facile Synthesis of Eggshell Membrane-templated Au/CeO_2_ 3D Networks for Nonenzymatic Electrochemical Dopamine Sensor**

Qingquan Liu^a#^, Xiaoyu Chen^a#^, Ze-Wen Kang^b^, Chaohui Zheng^a*^, Da-Peng Yang^a,b^*

^a^Department of Pulmonary and Critical Care Medicine, The Second Affiliated Hospital of Fujian Medical University, Quanzhou 362000, Fujian Province, China.

^b^College of Chemical Engineering and Materials Science, Quanzhou Normal University, Quanzhou, Fujian Province, China.

^#^ These authors contributed equally to this work.

*Corresponding authors, email: [yangdp@qztc.edu.cn](mailto:yangdp@qztc.edu.cn); [drzch91@163.com](mailto:drzch91@163.com)


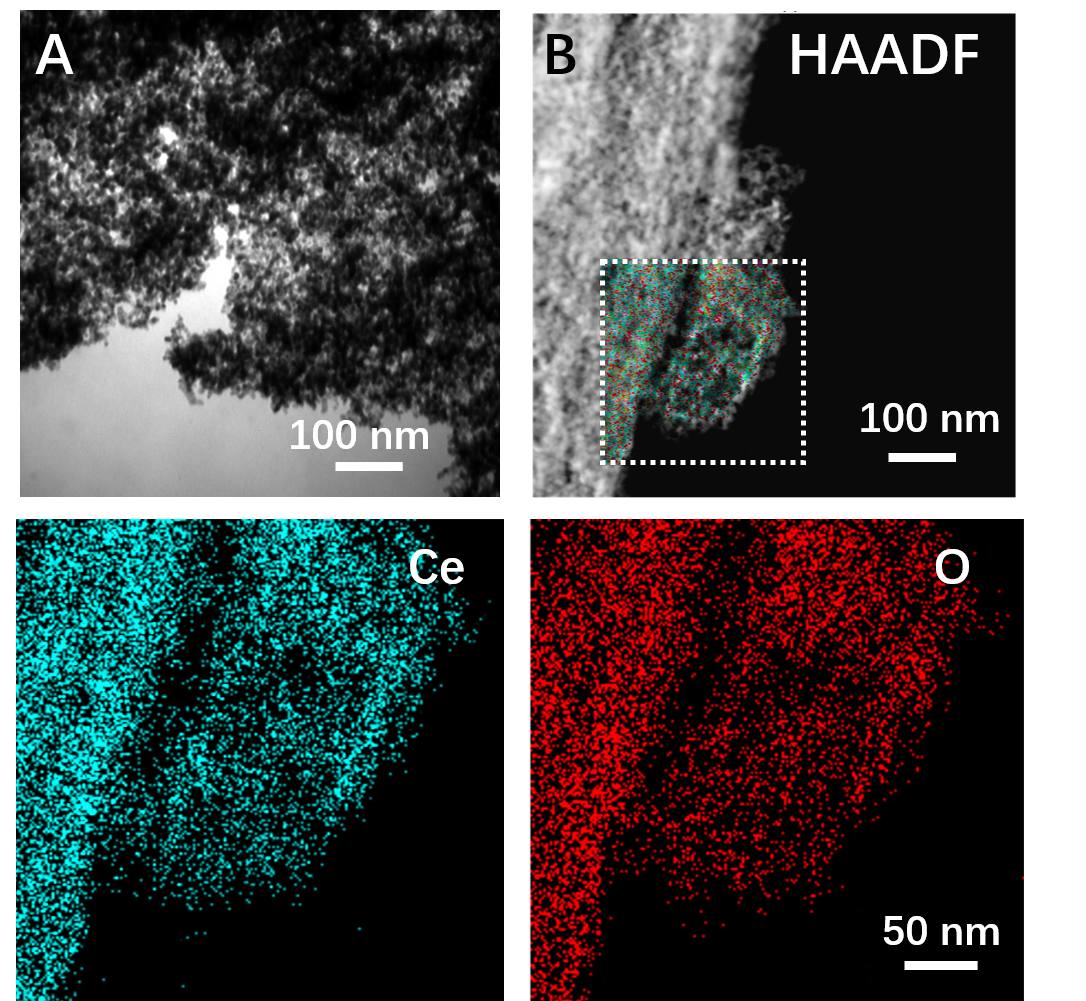


Figure S1. (A) TEM image of ESM-C nanocomposites and (B) TEM-based elemental mapping of ESM-C nanocomposites.


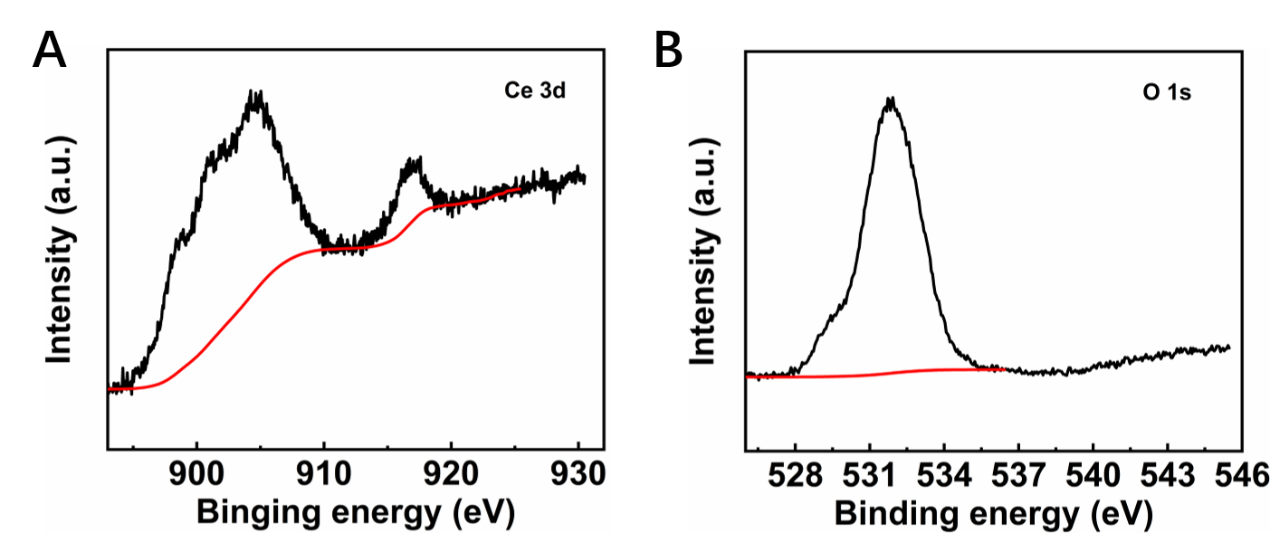


Figure S2. XPS spectra of ESM-C nanocomposites: (A) Ce 3d and (B) O 1s.


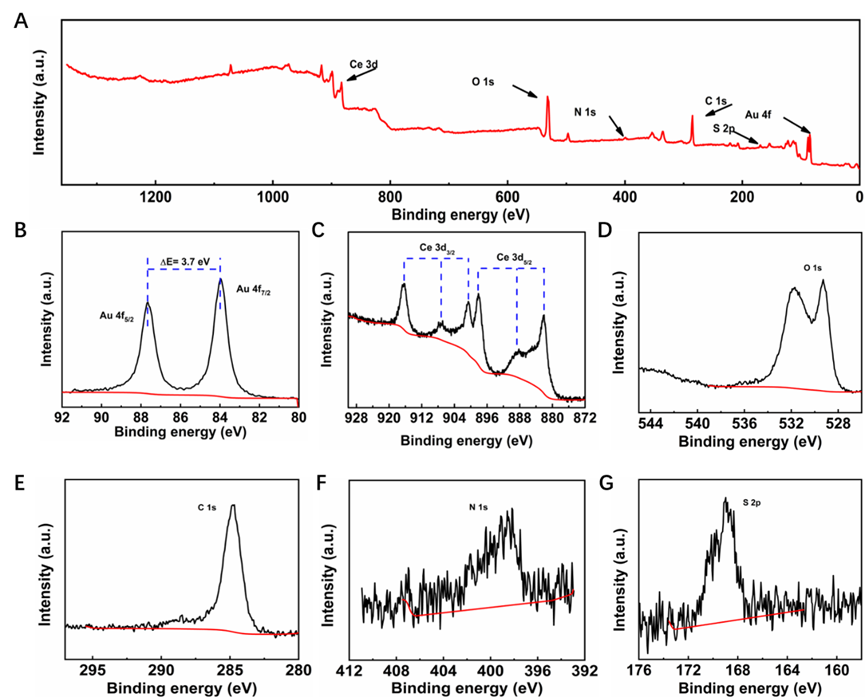


Figure S3. Full XPS spectra of ESM-AC nanocomposites.


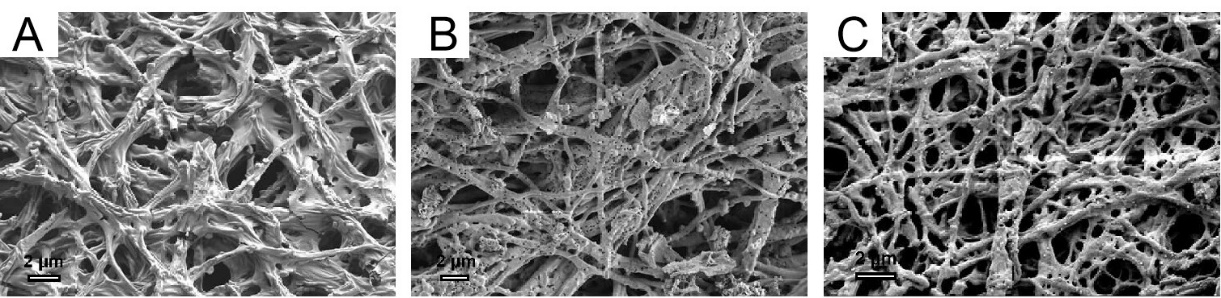


Figure S4. SEM images of (A) ESM; (B) ESM-C and (C) ESM-AC on the surfaces of electrodes.

**Table S1.** The analytical performances of various meterials for the detection Dopamine.

| **Sensors** | **Detection range (μM)** | **LOD (μM)** | **Reference** |
| --- | --- | --- | --- |
| CNTs-La(OH)_3_ | 0.5-35.4 | 1.67 | [^1^](#_ENREF_1) |
| Fc@DWNTs/GCE | 0.5-20 | 0.3 | [^2^](#_ENREF_2) |
| Ni/Al-LDH | 10-700 | 5.0 | [^3^](#_ENREF_3) |
| CILE | 2-150 | 1.0 | [^4^](#_ENREF_4) |
| Pristine Graphene | 5-710 | 2.0 | [^5^](#_ENREF_5) |
| ESM-AC | 100-10000 | 0.26 | **This work** |

**Reference**

1. Y. Zhang, R. Yuan, Y. Chai, X. Zhong and H. Zhong, *Colloids Surf., B*, 2012, **100**, 185-189.

2. H. Cheng, H. Qiu, Z. Zhu, M. Li and Z. Shi, *Electrochim. Acta*, 2012, **63**, 83-88.

3. Z. Zhu, L. Qu, Y. Guo, Y. Zeng, W. Sun and X. Huang, *Sens. Actuators, B*, 2010, **151**, 146-152.

4. A. Safavi, N. Maleki, O. Moradlou and F. Tajabadi, *Anal. Biochem.*, 2006, **359**, 224-229.

5. S. Qi, B. Zhao, H. Tang and X. Jiang, *Electrochim. Acta*, 2015, **161**, 395-402.
